# Summary of the effect of an exercise intervention on antenatal depression and the optimal program: a systematic review and Meta-analysis

**DOI:** 10.1186/s12884-023-05629-y

**Published:** 2023-04-26

**Authors:** Lanjuan Liu, Cheng Liu, Xiaotang Liu, Yang Yang

**Affiliations:** 1 College of Physical Education, Shanghai Normal University, Shanghai, 200234 China; 2 Department of Sports, Donghua University, Shanghai, 201600 China

**Keywords:** Antenatal depression, Exercise intervention, Effect size, Meta-analysis

## Abstract

**Objective:**

This study aimed to examine the effect of exercise intervention for antenatal depression using meta-analysis and to propose the best exercise intervention program.

**Methods:**

Review Manager 5.3 was used to analyze 17 papers with 2224 subjects by setting five moderators, including type, time, frequency, period, and format of exercise intervention, and a random-effects model was used to test for overall effect, heterogeneity, and publication bias.

**Results:**

(1) The effect size of the exercise intervention on antenatal depression was d = -0.56, which reached a good effect and was statistically significant; b (2) The effect size of the exercise type on antenatal depression was Yoga and a combination of aerobic exercise in order of intervention; (3) the single intervention duration of 10–75 min all had a good effect on antenatal depression, and 30–60 min had the best effect; (4) the intervention frequency of 3 to 5 times/week had the greatest amount of intervention effect on maternal depression; (5) exercise lasting 6–10 weeks had a good intervention effect on antepartum depression, and the amount of effect decreased gradually with the extension of time; (6) In terms of exercise format, the amount of intervention effect on maternal depression was in the order of group exercise, individual + group exercise.

**Conclusions:**

Exercise intervention can significantly alleviate antenatal depression symptoms. The best exercise program for exercise intervention for antenatal depression is: Yoga and a combination of aerobic exercise intervention effects are more prominent, and the intervention effect of Yoga is the best. The use of group exercise 3–5 times per week for 30–60 min for 6–10 weeks was more likely to achieve the desired intervention effect of improving antenatal depression.

## Introduction

Depression is the most common psychiatric disorder in the general population, with a prevalence twice as high in women as in men and an exceptionally high risk in women of childbearing age [1]. Antenatal depression may negatively affect the physical and mental health of both mother and fetus [2], with children of depressed mothers having lower birth weight, higher risk of resting heart rate, developmental delay, and premature birth, greater physiological reactivity, and more behavioral problems in childhood and adolescence than children of non-depressed mothers [3]. In addition, antenatal depression is considered the strongest risk factor for postpartum depression and mediates the relationship between risk factors and postpartum depression [4]. Globally, 20.7% of women are affected by antenatal depression [5], and 14.3% of women in China have experienced antenatal depression. The incidence of antenatal depression among Chinese women has been increasing rapidly yearly due to the increase in older women, menstruating mothers, and women with underlying diseases [6]. Exercise therapy is a treatment that causes changes in the human body based on exercise physiology and other factors to improve somatic physiological, psychological, and mental dysfunction [7]. Exercise is a non-invasive, low-cost alternative therapy for relieving mental illness, especially favored by patients with antenatal depression, as it can be anti-depress, regulate the neuroendocrine system, and increase the concentration of monoamine neurotransmitters in the brain [8]. However, there is a lack of international standards for exercise guidelines related to interventions for antenatal depression, and the participation rate in exercise among pregnant women is low. Choosing the appropriate type, intensity, duration, and frequency of exercise is the primary key to enhancing the efficacy of exercise for antenatal depression [9].

Studies on the effect of exercise on antenatal depression have focused on intervention experiments and review analyses. Experimental studies have mainly explored the intervention effects of different exercises on antenatal depression, but various exercise intervention protocols showed multiple and significant differences, and the usefulness for practice needs to be improved [9]. The review studies generally focused on analyzing the causes and mechanisms of improvement of antenatal depression by exercise interventions in the literature, with the little specific exploration of the design of exercise protocols. The existing systematic reviews and meta-analyses have mainly focused on exploring the effect sizes of exercise interventions on antenatal depression, with few studies on the settings of modifying core variables such as types and frequencies of exercise in exercise intervention programs and their associations with antenatal depression. The lack of studies directly leads to the lack of guiding recommendations for existing exercise programs for antenatal depression. The effectiveness of exercise interventions for antenatal depression can only be further improved based on the development of precise exercise protocols [10]. To investigate the effect of an exercise intervention on prenatal depression and the optimal protocol, we used evidence-based medicine as the guiding principle to analyze the published randomized controlled trials on exercise intervention for prenatal depression and to evaluate the overall effect of an exercise intervention on prenatal depression. We also investigate the association between factors such as exercise protocol and the overall effect to identify the optimal exercise type, exercise frequency, single exercise time, duration, and exercise form and then to refine the optimal exercise protocol for exercise intervention for prenatal depression, to provide evidence and reference for clinical treatment of exercise intervention for prenatal depression.

## Data and methods

### Literature search

Following the principle of convenient search, and due to the full liberalization of the birth policy and the release of fertility demand along with the three-child policy, China has become one of the countries with great international attention to exercise for antenatal depression. Therefore, this study searched databases, including Chinese databases and foreign language databases. The Chinese search databases included the Chinese National Knowledge Infrastructure (CNKI), Wanfang Data, and VIP Database, the foreign language search databases included Cochrane Library, SCI, PubMed, Medline, and Embase. Retrieval strategies were mainly based on MeSH subject words and free words.In addition,the search period was from the creation date of the databases to August 2022, and the references to the retrieved literature were traced.Taking PubMed as an example, the retrieval strategy is shown in Table 1.

**Table 1:** Search strategy for PubMed database

Search	Query
#1	Pregnant Women[MeSH Terms]
#2	“Pregnant Women“[MeSH Terms] OR “pregnant woman“[Title/Abstract] OR “woman pregnant“[Title/Abstract] OR “women pregnant“[Title/Abstract]
#3	Depression[MeSH Terms]
#4	“Depression“[MeSH Terms] OR “depressive symptoms“[Title/Abstract] OR “depressive symptom“[Title/Abstract] OR “symptom depressive“[Title/Abstract] OR “emotional depression“[Title/Abstract] OR “depression emotional“[Title/Abstract]
#5	“Exercise“[MeSH Terms]
#6	Exercise[MeSH Terms] OR “Exercises“[Title/Abstract] OR “physical activity“[Title/Abstract] OR “activities physical“[Title/Abstract] OR “activity physical“[Title/Abstract] OR “physical activities“[Title/Abstract] OR “exercise physical“[Title/Abstract] OR “exercises physical“[Title/Abstract] OR “physical exercise“[Title/Abstract] OR “physical exercises“[Title/Abstract] OR “acute exercise“[Title/Abstract] OR “acute exercises“[Title/Abstract] OR “exercise acute“[Title/Abstract] OR “exercises acute“[Title/Abstract] OR “exercise isometric“[Title/Abstract] OR “exercises isometric“[Title/Abstract] OR “isometric exercises“[Title/Abstract] OR “isometric exercise“[Title/Abstract] OR “exercise aerobic“[Title/Abstract] OR “aerobic exercise“[Title/Abstract] OR “aerobic exercises“[Title/Abstract] OR “exercises aerobic“[Title/Abstract] OR “exercise training“[Title/Abstract] OR “exercise trainings“[Title/Abstract] OR “training exercise“[Title/Abstract] OR ”Trainings, Exercise”[Title/Abstract])
#7	“Randomized Controlled Trial“[Publication Type]
#8	#2 AND #4 AND #6 AND #7

### Inclusion and exclusion criteria

#### Inclusion criteria

According to the PICOS principles of Cochrane systematic evaluation, the literature inclusion criteria included (1) type of literature: randomized controlled trials; (2) study population: the entire sample met the diagnostic criteria for depression in the structured clinical interview for depression, (not taking antidepressant-type therapeutic drugs) singleton pregnancy, and no other pregnancy complications; (3) type of intervention: the experimental intervention method was exercise (yoga, maternal health care exercise, fertility dance), the control group was irregular exercise, including waiting list control, daily care or active control (such as health education); (4) inclusion indexes and measures: antenatal depression rating scale; (5) trial data processing: the trial data contained effect sizes and standard deviations for pre-test, post-test or pre- and post-test comparisons.

#### Exclusion criteria

The studies about (1) depression due to alcoholism or non-alcoholic psychoactive substances, (2) depressive episodes due to physical illness, (3) not containing available outcome information, (4) not written in Chinese or English, (5) unpublished papers, and (6) animal studies were excluded.

### Data extraction

Data extraction and input were done independently by the evaluators, excluding studies that did not meet the criteria, and literature that might meet the inclusion criteria was read in its entirety to determine whether the inclusion criteria were met. Literature information was collected using our self-administered form, including authors, year of publication, the total number of study subjects, the sample size of intervention and control groups, week of pregnancy, type of exercise intervention, frequency, duration, the format of exercise, and method of measurement.

### Quality assessment of the literature

The literature’s methodological quality was evaluated using the Cochrane Risk Assessment Tool, mainly in 6 aspects: selection bias, performance bias, detection bias, follow-up bias, reporting bias, and other biases. The quality was also scored (out of 7) according to 3 levels (low risk, high risk, and unclear), and the quality of the included literature was classified into three levels from high to low: high quality (5 and above), moderate quality (3–4), and low quality (2 and below).

### Statistical analysis

A meta-analysis of the outcome indicators of the included literature was performed using RevMan 5.3 software, and the measures were expressed as mean difference (MD) and its 95% confidence interval (CI), and the intervention effects were expressed as mean ± standard deviation (SD). The I^2^ statistic was used to test for heterogeneity among studies, and I^2^ ≤ 50% indicated no heterogeneity among similar studies, and the fixed effect model was selected for the combined statistical analysis. I^2^ ˃ 50% showed heterogeneity among studies, and then the random-effects model was used to combine the effects size, and further subgroup analysis was conducted to find the source of heterogeneity [10].

## Results

### Literature search results

**Fig. 1:**
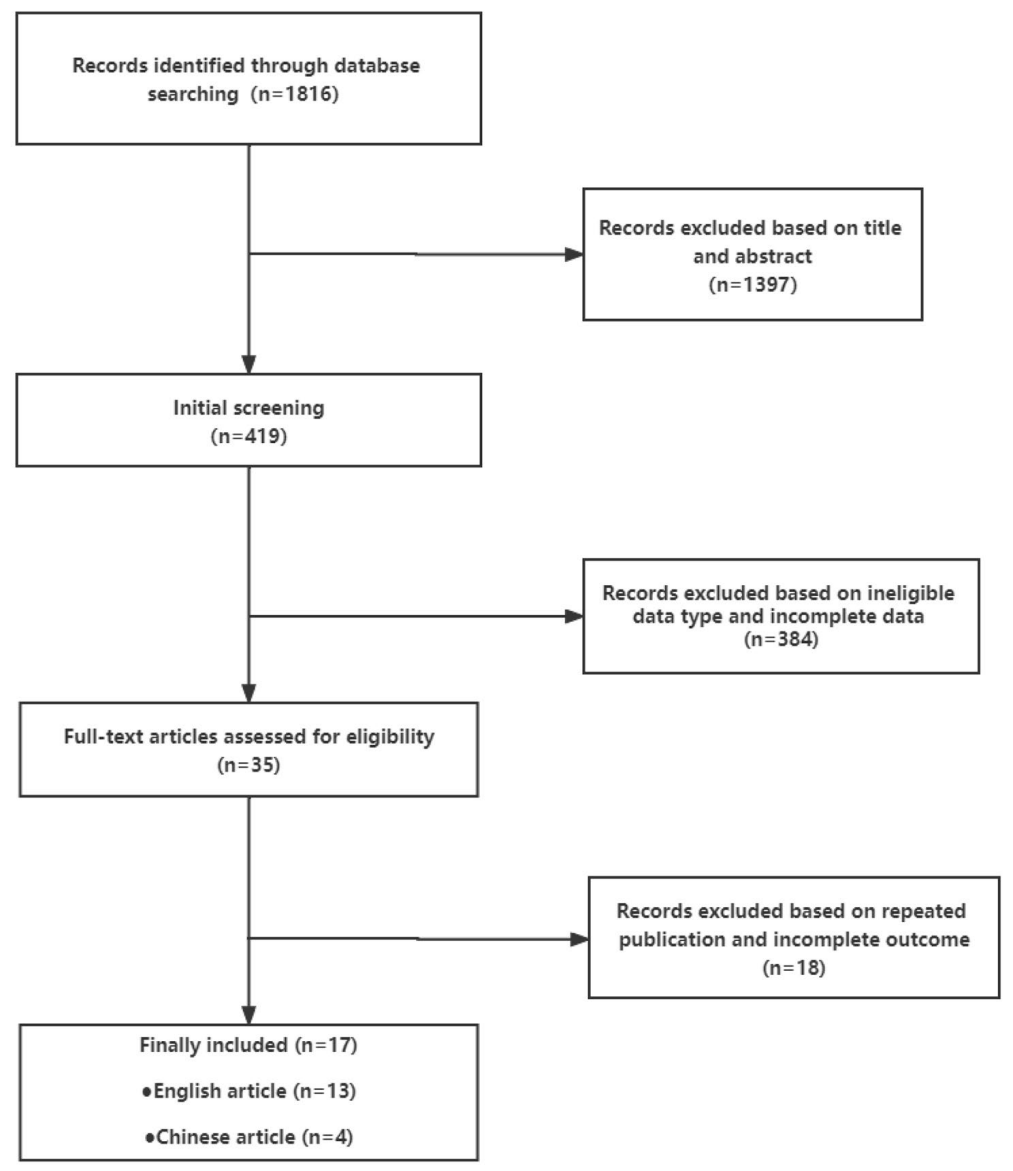
Flowchart of Literature Search and Study Selection

A total of 1816 papers related to the topic of this study were retrieved from eight databases, and 419 articles were obtained by title screening and initial abstract screening. Then 35 potentially relevant papers were obtained by reading the complete text, excluding documents with incomplete data and those reporting only outcome change values. Finally, after screening for duplicate publications and incomplete indicators, 17 papers were included, including 13 in the foreign language and 4 in Chinese (Fig. 1).

### Characteristics of the included studies

There were 17 randomized controlled trials and 1,463 subjects with singleton pregnancies with pregnancy weeks ranging from 9 to 28, more often concentrated in 12–22 weeks. The types of interventions in the exercise intervention program were mainly moderate-intensity Yoga, the combination of aerobic exercise, dance, and maternal health care exercise. The intervention protocols varied considerably between studies. The duration of single interventions ranged from 10 to 75 min, with a higher concentration of 30–60 min. The frequency of interventions ranged from 1 to 5 times per week, three times per week being the most frequent. The intervention period ranged from 8 to 30 weeks, with 12 weeks being the most frequent. The format of exercises included group exercises and individual combined with group exercises. The control group usually used conventional antenatal education and did not receive mental health education. Ending indicators included the Edinburgh Postnatal Depression Scale (EPDS), the Center for Epidemiologic Studies Depression Scale (CES-D), or other depression measurement scale assessments that reflect depression status. The basic characteristics of the included studies are shown in Table 2.

**Table 2:** Characteristics of Studies Included in Meta-analysis

First author	Sample information		Intervention method					Outcome Indicator
	Total (intervention group/control group)	Intervention time point	Intervention in the experimental group	Intervention in the control group	Intervention exercise frequency (min/week)	Intervention duration (weeks)	Exercise format	
Haiying Zhang (2016)	77 (38/39)	18-22weeks	Yoga	Antenatal care	2 times/week, 75 min/ time	8 weeks	Group	CES-D, STAI
Tiffany F (2012)	84 (42/42)	18–22 weeks	Yoga	Antenatal care	2 times/week, 20 min/ time	12 weeks	Group	CES-D, STAI, STAXI,
Tiffany F (2013)	92 (46/46)	Means 22 weeks	Tai Chi and Yoga	Waiting list control group	1 time/week, 20 min/time	12 weeks	Group	SCID, CES-D, STAI, Sleep Dysfunction Rating Scale
M.Satyapriya (2013)	96 (45/51)	20–36 weeks	Yoga	Antenatal education	3 times/week, 60 min/ time	16 weeks	Group	PEQ, STAI, HADS
Kyle D (2015)	39 (20/19)	28 weeks	Yoga	Conventional treatment	1 time/week, 75 min/ time	8 weeks	Group	EPDS, STAI-S, STAI-T, PANAS-N
Ling Shu (2019)	78 (40/38)	10–28 weeks	Yoga	Usual psychological care	1 time/week, 60 min/ time	6 weeks	Group	SAS, EPDS
Jennifer M (2012)	24 (12/12)	20 weeks	Yoga	Parenting Education	2 times/week, 20 min/ time	12 weeks	Group	CES-D
Lisa A U (2016)	18 (11/7)	12–26 weeks	Yoga	Health education	75 min/ time	9 weeks	individual + Group	QIDS, EPDS
Feng Yan (2019)	212 (101/111)	-	Dance	Health education	3–4 times/week, 30-45 min/time	24 weeks		EPDS
M Perales (2015)	167 (90/77)	9–12 weeks	Dance	Antenatal care	3 times/week, 55-60 min/time	About 30 weeks	Group	CES-D
Marina V-T (2017)	124 (70/54)	< 16 weeks	Exercise	-	3time/ weeks, 60 min/time	24–27 weeks	Group	CES-D
Xinhua Liu (2016)	137 (67/70)	20 weeks	Maternal health care exercise	-	3–5 times/week, 10-15 min/time	24 weeks	Group	SAS, CES-D
Ellahe S (2016)	30 (15/15)	-	Yoga	-	-	8 weeks	Group	Hamilton Rating Scale for Depression, Perceived Stress Scale, and Inventory of Subjective Life Quality
Liu Rong (2020)	64 (32/32)	18–27 weeks	Yoga	Usual care	3 times/week, 60 min/time	12 weeks	Group	EPDS
Marina Vargas-Terrones (2021)	61 (36/25)	<16 weeks	Combination of Aerobic exercise Combination of Aerobic exercise	Personal Care	4 times/week, 60minIn/time	24–26 weeks	Group	CES-D
Mervat M (2016)	100 (50/50)	Second trimester	Combination of Aerobic exercise	Usual care	3 times/week, 60Min/time	12 weeks	Group	CES-D
Reza nikbakht (2016)	64 (32/32)	16–20 weeks	Combination of Aerobic exercise	Usual care	3 times/week, 30–45 min/time	8 weeks	Group	Beck Depression Inventory (2nd version)

### Assessment of the methodology of the included literature

**Fig. 2:**
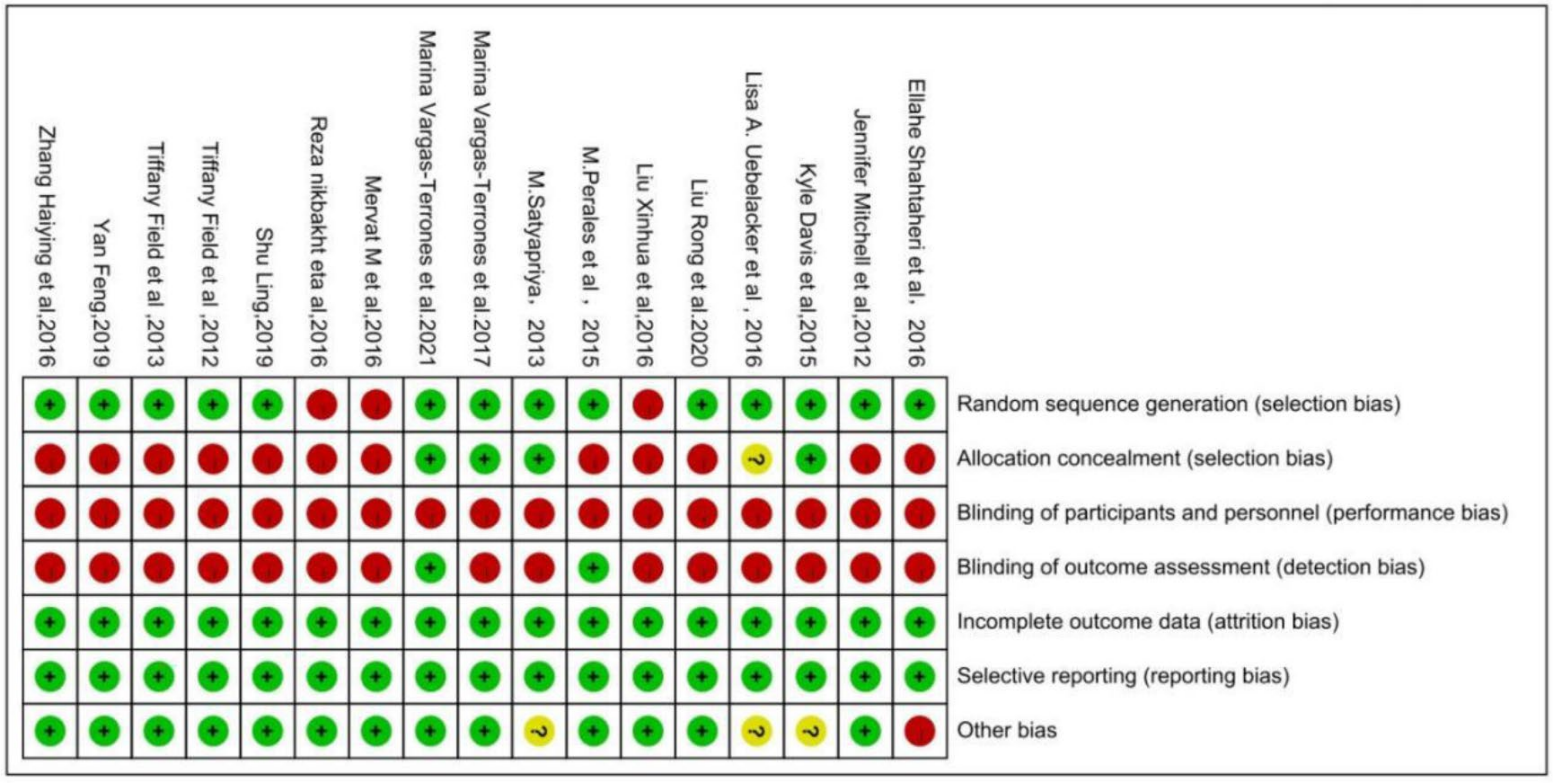
Methodological Quality of Included Studies. Note: Categories: “+” met the standard, and “-“ did not meet the standard

**Fig. 3:**
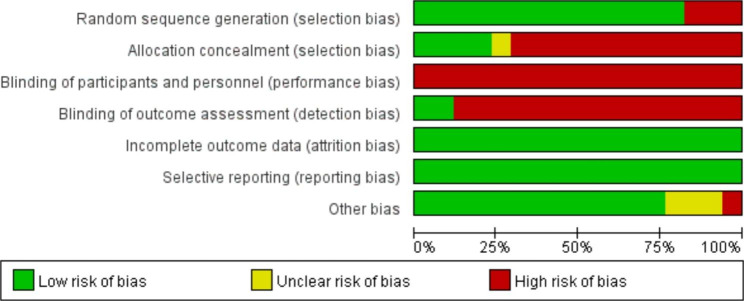
The Distribution of the Methodological Quality of Included Studies

The difficulty of implementing a double-blind trial of an exercise intervention for patients with antenatal depression impacts the quality of the literature, leading to a high degree of heterogeneity when doing a meta-analysis. The methodological assessment of the literature’s quality is shown in Fig. 2, indicating that three papers reached a low risk of bias and were of high quality, with two of them reaching a score of 6. The remaining 14 papers met the moderate risk of bias. Those that met the standard were labeled as “+,“ and those that did not were labeled as “-“ (Fig. 2). Figure 3 shows the statistical plot of the percentage of each entry of the methodological assessment. The included literature was generally not blinded to the experimental implementer. Detailed knowledge of the purpose of the experiment by subjects and experiment implementers facilitates the smooth implementation of the intervention and the scientific monitoring of the experimental process. The exercise improved antenatal depression mainly through a series of mediators. Therefore, the lack of blinding of subjects and implementers did not affect the effect of exercise on the improvement of antenatal depression.

### Test for publication bias

**Fig. 4:**
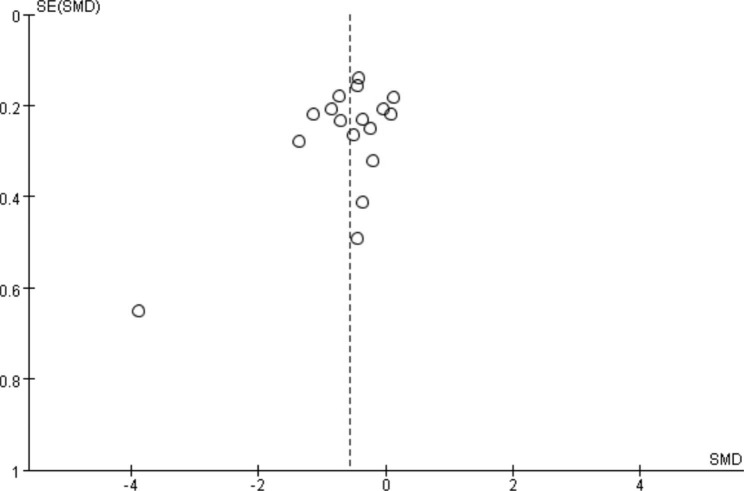
Funnel Plot of Exercise Intervention in Relieving Antenatal Depression

Funnel plots are initially scatter plots using the treatment effect estimates for each study as the X-axis and the sample content size as the Y-axis to observe the publication bias of articles. Funnel plots are inappropriate if the literature is too small and are usually required when the number of studies in a Meta-analysis is ten or more [11]. The number of studies included in this study was 17, allowing for a test for publication bias. Figure 4 reveals that one piece of literature has some distance bias from the rest, indicating heterogeneity. In addition, the scatter distribution is on the upper X-axis with a largely symmetrical left-right distribution, showing no significant publication bias.

### Sensitivity analysis

Sensitivity analysis is a method used to evaluate whether the results of a meta-analysis or systematic review are stable and reliable. Sensitivity analysis was performed on the 17 included papers, mainly by excluding documents one by one and changing the analysis model, and on this basis, the effect size was re-examined by calculation. The test found that the results did not vary significantly, indicating that the results of the Meta-analysis of this study were credible.

## Results of the meta-analysis

### Test for overall effect test of the intervention effect

After an overall intervention effect size test on the entire sample of 17 papers included in the study, it was found that exercise positively improved antenatal depression (Table 3). The test for overall heterogeneity (I^2^ = 79%, p < 0.00001) was performed on the included literature, indicating heterogeneity among multiple studies, and the effect sizes were combined using a random effects model. The heterogeneity among multiple data sets in this Meta-analysis reflects the possibility of multiple moderating variable factors affecting the overall effect size.

**Table 3:** Statistical Analysis of the Association of Exercise Intervention with Antenatal Depression

Number of literature	Heterogeneity test			Effect size and 95% confidence interval	Two-tailed test	
	χ^2^	P	I^2^		Z	P
17	77.10	< 0.00001	79%	-0.56 (-0.81, -0.32)	4.51	< 0.00001

**Fig. 5:**
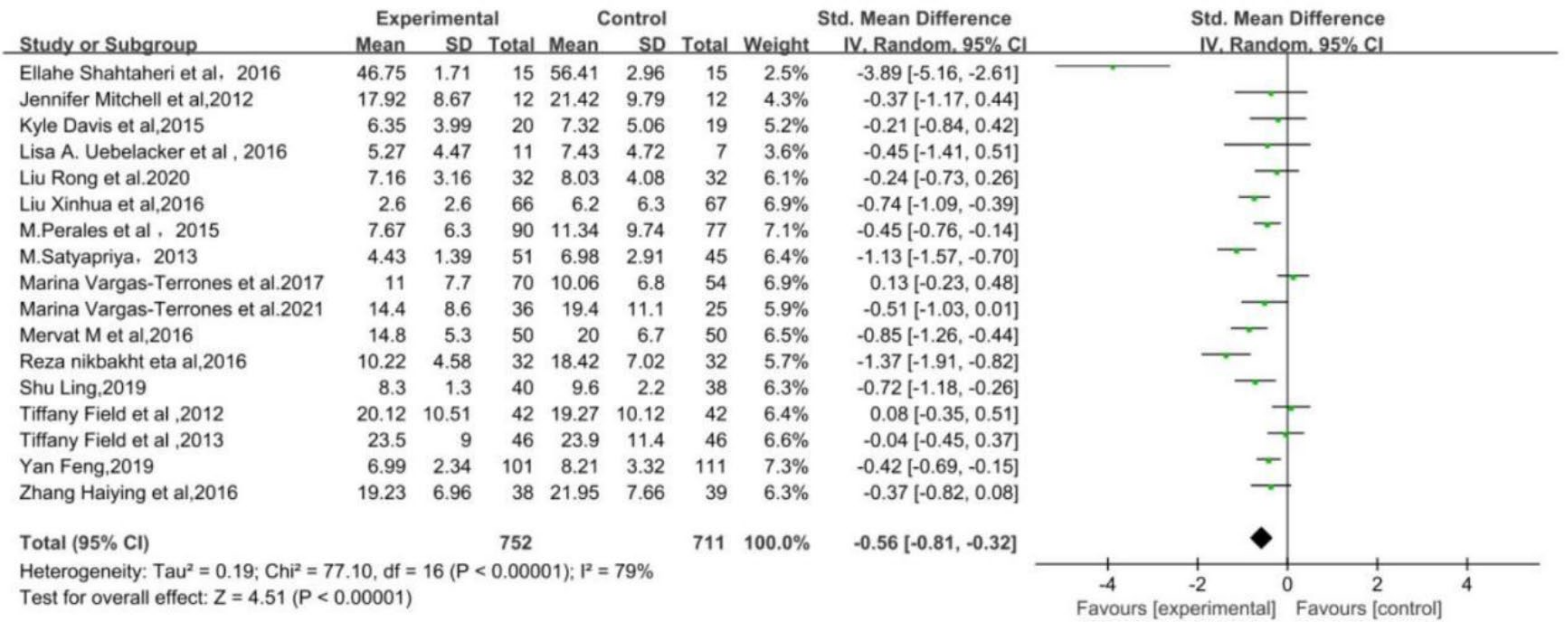
Meta-analysis of the Association Between Exercise Intervention and Antenatal Depression. Note: The “-“ before the effect size represents the direction of the effect size, and “-“ represents a decrease in depression scale score, i.e., depression is relieved. Experimental: experimental group; control: control group; Mean: mean value; SD: standard deviation; Total: sample size; SMD: standardized mean difference; Random: random effects model

The “-“ in front of the effect size indicates that exercise can alleviate antenatal depression. Figure 5 shows that the standardized mean difference of exercise intervention for antenatal depression was d = -0.56 (p < 0.00001). Cohen’s (1988) study explains that 0.2 is a small effect, 0.20 < |d| < 0.80 is a medium effect, and 0.8 or more is a significant effect [12], indicating that exercise improved antenatal depression up to a medium effect size. The results of the two-tailed test (P < 0.00001) showed that the combined statistics of multiple data sets were statistically significant with a 95% confidence interval of (-0.81, -0.32). The above data indicated that the exercise intervention positively improved antenatal depression.

### Subgroup analysis of moderators

Potential moderators were possible based on the test for overall effects. Therefore, the present study examined the moderating variables affecting the relationship between exercise intervention and prenatal depression (Table 4).

**Table 4:** Moderating Effect Test of Meta-analysis of the Association between Exercise Intervention and Antenatal Depression

Moderators	Homogeneity test			Category	Effect size and 95% CI	Two-tailed test		Number of literature	Number of samples
	X^2^	P	I^2^(%)			Z	P		
Intervention Content				Yoga	-0.59 (-1.00, -0.17)	2.78	0.005	10	602
				Combination of Aerobic exercise	-0.58 (-1.04, -0.13)	2.52	0.01	5	516
	77.10	< 0.00001	79	Dance	-0.42 (-0.69, -0.15)	3.02	0.003	1	212
				Maternal health care exercise	-0.74 (-1.09, -0.39)	4.13	0.56	1	133
Duration				10–30 min	-0.27 (-0.70, 0.17)	1.19	0.23	4	333
	49.48	< 0.00001	70	30–60 min	-0.60 (-0.88, -0.32)	4.18	< 0.00001	9	966
				60–75 min	-0.33 (-0.67, 0.01)	1.90	0.06	3	134
Frequency				1 time/week	-0.34 (-0.69,0.02)	1.85	0.06	4	227
	49.48	< 0.00001	70	2 times/week	-0.17 (-0.48, 0.15)	1.04	0.30	3	185
				3–5 times/week	-0.60 (-0.88, -0.33)	4.29	< 0.00001	9	1021
Weeks				6–10 weeks	-1.20 (-2.04, -0.36)	2.79	0.005	6	306
	83.17	< 0.00001	82	12–16 weeks	-0.41 (-0.71, -0.11)	2.71	0.007	8	865
				24–30 weeks	-0.26 (-0.68,0.15)	1.25	0.21	3	352
Exercise format				Group	-0.60 (-0.89, -0.31)	4.05	< 0.00001	16	1251
	76.84	< 0.00001	80	Group + individual	-0.42 (-0.69, -0.15)	3.02	0.003	1	212
				Individual	-	-	-—	0	0

By subgroup analysis of intervention content, the four groups were highly heterogeneous in terms of differences in effect sizes (I^2^ = 79%), indicating an effect of the exercise program on the relationship between exercise intervention and antenatal depression. Yoga (d = -0.59, p = 0.005) and a combination of aerobic exercise (d = -0.58, p = 0.01) had comparable effects, and the p-values of effect sizes for these two groups were < 0.05 and significant, while dance and maternal health care exercise both had only one literature, which was not representative enough to include a total of 1463 cases.

By subgroup analysis of exercise duration, the three groups were highly heterogeneous regarding effect size differences (I^2^ = 70%), indicating an effect of exercise duration on the relationship between exercise intervention and antenatal depression. The largest effect size was found for 30–60 min (d = -0.06), which reached a moderate effect size, and most of the literature was enriched in this group, followed by 60–75 min (d = -0.33, p = 0.006). 10–30 min had the smallest effect size (d = -0.27, p = 0.23), with 1433 subjects included in the study.

By exercise frequency subgroup analysis, the three groups were highly heterogeneous (I^2^ = 82.5%) for alleviating antenatal depression, indicating the effect of exercise frequency on the relationship between exercise intervention and antenatal depression. The 3–5 times/week group produced the largest effect size (d = -0.60, P < 0.00001), followed by the once/week group (d = -0.34, P = 0.06), and finally, the two times/week group (d = -0.17, P = 0.30), with a total of 1433 study subjects included.

By exercise cycle subgroup analysis, the three groups were highly heterogeneous (I^2^ = 82%) for alleviating antenatal depression, indicating an effect of the exercise cycle on the relationship between exercise intervention and antenatal depression. The largest effect size was produced by 6–10 weeks of exercise (d = -1.20, P = 0.005), which was significantly better than the other groups, followed by the 12–16 weeks group (d = -0.41, P = 0.007), and the smallest was the 24–30 weeks group (d = -0.26, P = 0.21), with a total of 1523 subjects included in the study.

By subgroup analysis of exercise format, the two groups were highly heterogeneous (I^2^ = 80%) for alleviating antenatal depression, indicating an effect of exercise format on the relationship between exercise intervention and antenatal depression. Due to the particularity of pregnant women, most of the literature is on group exercise, producing a large effect size (d = -0.60, P < 0.00001). There was only one article on individual + group exercise, which was not representative enough, and this meta-analysis lacked individual exercise, which is an imbalance, with a total of 1463 cases included in the study.

## Discussion

### Analysis of the overall effect of the included literature

Due to the specificity of pregnant women and the implementation of exercise monitoring, the subjects and implementers were not double-blinded, affecting the literature quality but did not affect the effect of exercise on the improvement of prenatal depression. The results of this Meta-analysis to assess the quality of the literature of the 17 included papers showed that the highest quality score was six and the lowest was 3. The literature with lower scores was mainly related to the absence of detailed descriptions of random assignment, detailed inclusion criteria for study subjects, blinded design, and detailed reporting of factors such as subject dropouts.

Publication bias results showed that the 17 included papers were largely symmetrically distributed between left and right, indicating high stability. Analysis revealed a moderate effect size (P < 0.000 01) and 95% confidence interval (-0.81, -0.32) for the combined effect size of exercise interventions for prenatal depression, suggesting that exercise interventions are effective in improving prenatal depression. Two studies conducted a meta-analysis of Yoga to improve antenatal depression, and both reported a significant intervention effect, providing empirical data to support the results of this study [13, 14]. Fifteen of the 17 studies in the test for overall effect had a negative effect size, indicating that the effect of alleviating antenatal depression was achieved. Of course, a few prior studies were inconsistent with the results of this study, such as the study by Smith C A et al., which pointed to no evidence that Yoga reduced depression in their systematic evaluation, which the authors analyzed as the reason for this is that high-quality randomized controlled trials still need to be included [15].

### Analysis of the effect of exercise program moderators on antenatal depression

Because of the high overall heterogeneity of exercise interventions for antenatal depression (I^2^ = 79%), this study introduced moderators to investigate the heterogeneity in depth. When the relationship between variable Y and variable X is a function of variable M, the relationship between Y and X is influenced by a 3rd variable-M, and the M variable is the moderating variable [16].

### Intervention content

This study found that the maternal health care exercise group produced the largest effect size and had the best effect in improving antenatal depression. However, the conclusions may be somewhat biased because only one piece of literature was included in this group, and its findings were not significantly different. The moderate-intensity Yoga and the combination of aerobic exercise groups had comparable intervention effects, but the yoga group included more literature and had a larger sample size. It was also found that women liked Yoga the most during pregnancy, which had high reliability and satisfaction as an intervention for depression during pregnancy. Another study has revealed less depression and anger in the yoga group compared to the social support group. Yoga, as a form of self-massage, has similar efficacy to massage in relieving indicators of depression [17]. Yoga has efficacy as a monotherapy for major depression or an anti-depressant augmentation. It has anti-depressant effects comparable to imipramine and is more effective than other interventions, such as massage and walking [18]. In summary, it can be concluded that the moderate-intensity yoga group was superior to other exercise formats in the study.

Yoga is a safe, gentle, and particularly helpful form of breathing, asana, meditation, and relaxation tailored for pregnant women and is a promising strategy for treating antenatal depression. The negative correlation between Yoga and depression can be explained by the following six mechanisms. (i) Modulation of neuroendocrine system function: Yoga enhances parasympathetic tone, and increased parasympathetic activity may lead to reduce the discharge of the lateral nucleus reticular is paragiagan to cellularis to the blue spot, which reduces norepinephrine output, resulting in relaxation, calmness, and slower breathing and heart rate [19]. Yoga may activate the prefrontal cortex and enhance glutamatergic transmission in the medial hypothalamic arcuate nucleus, releasing β-endorphins and decreasing cortisol [20]. (ii) Increased stress tolerance: yoga breathing decreases chemical reflex sensitivity, improves baroreflex sensitivity, and increases exercise and stress tolerance. (iii) Modulation of autonomic nerve changes: Yoga reduces HPA and SAM hyperactivity, decreases sympathetic arousal and enhances parasympathetic tone [21]. Yoga interventions improve Autonomic Nervous System (ANS) stability and plasticity, thus enabling pregnant women to return to a calmer state more quickly after facing adverse emotions [22]. (iv) Adjustment of the balance of the brain’s cortical areas: Yoga may modulate top-down inhibition of amygdala activity and activation of the anterior cingulate cortex. Yoga encourages positive thinking, positive self-talk, and self-acceptance, helping increase self-confidence and self-awareness by encouraging focus on physical movement, breathing, and meditation to activate the ventral medial prefrontal cortex (VMPFC) [23]. (v) Increase in brain neurotransmitters: yoga practice has the potential to act on neurotransmitters, especially at the synapses. It increases monoamine neurotransmitters such as dopamine (DA) and gamma-aminobutyric acid (GABA). Yoga breathing exercises stimulate the vagus nerve, and the deep and slow breathing pattern has been shown to enhance prolactin, dopamine, norepinephrine, and serotonin [24]. (vi) Improved stress adaptation: yoga asanas and breathing exercises modulate stress-responsive brain regions, improve HPA axis activity, autonomic balance, and inflammation, and reduce the drive of bottom-up stress pathways. Yoga’s regulation of emotions in terms of positive thoughts encourages positive coping, mediated by structures in the brain’s prefrontal cortex, thereby reducing the top-down stress drives [25]. Yoga may be effective for the short-term relief of depression by enhancing the overall regulation of the stress response system, thereby relieving depression [26].

### Duration of single practice, frequency of practice, and periodicity of practice in exercise programs

Pregnancy changes women’s body morphology, physiology, biomechanics, and psychology. These factors should be considered when developing the frequency, duration, and exercise cycle of exercise intervention programs, and thus making reasonable adjustments to exercise methods [27] to avoid additional fatigue and stress in pregnant women due to excessively long exercise [9] and to make exercise interventions safe and comfortable. Analysis of the included literature showed that the maximum amount of intervention effect was obtained for a single duration of 30–60 min, and a larger amount of intervention was maintained for 60–75 min and 10–30 min of exercise. Even a single short exercise of 10 min had a positive effect on antenatal depression and achieved an improvement in mood. Pregnant women without contraindications to exercise should be encouraged to participate in making exercise part of a healthy lifestyle. The greatest intervention effect was obtained with an intervention frequency of 3–5 times/week, followed by one time/week, and finally, two times/week. Andreas N et al. stated that it was due to the limited compliance with the twice-weekly intervention, but the treatment effect was positively correlated with the total number of hours of exercise, with the higher the anxiety level, the greater the benefit [28]. A study on the correlation between exercise frequency and depression in pregnant women found that prenatal depressive symptoms increased with decreasing exercise frequency and that there was a negative correlation between them [9]. It is consistent with the US ACOG recommendation of at least 150 min of moderate intensity for pregnant women at various times of the week, with 30–60 min of exercise per session.

The maximum prenatal anti-depressant intervention effect was achieved with exercise lasting 6–10 weeks, and the amount of intervention effect decreased gradually with longer intervention gestational weeks. Related studies have shown that exercise in early pregnancy may lead to threatened abortion due to embryonic instability. While in late pregnancy, due to weight gain and heavy body burden, exercise tends to overwork the body and may lead to adverse outcomes such as premature birth, so the mid-pregnancy stage of 6–10 weeks is the best intervention stage and gestational weeks.

Overall, under the premise of highlighting the principles of maximum caution and progressive load, it is important to focus on controlling the total intensity of exercise and adequate rest and recovery after exercise for pregnant women to avoid the accumulation of exercise load that causes adverse physical and psychological reactions or signs of excessive exercise load (e.g., over fatigue and sleep disorders), which in turn can increase the psychological burden and aggravate depression in pregnant women [9].

### Exercise format

Based on the safety of individual exercise for pregnant women, the lack of exercise monitoring, and technical support, exercise interventions were based on group and individual + group exercises. The group exercise format achieved the greatest amount of effect in improving antenatal depression. Group exercise promotes socialization and increases pregnant women’s motivation, exercise volume, and self-efficacy. Social support and communication are effective ways for pregnant women to overcome their bad moods, and the degree of social support is negatively associated with antenatal depression [9]. Group exercise interventions can increase communication and contact between pregnant women and pregnant women and pregnant women and health care providers, build maternal confidence in natural childbirth, prepare the mind for childbirth physiologically and psychologically, and eliminate triggers for the development of depression [29]. Regular group exercise reduces maternal fatigue, negative emotions, and common pregnancy symptoms (nausea/vomiting), as opposed to exercise alone [9]. In addition, group exercise may help improve the mastery of exercise intensity, the standardization of movements, and the accuracy of performing exercises as required, which to some extent, alleviates anxiety and depressive symptoms in pregnant women [29].

## Conclusions and recommendations

### Conclusion

Through literature review and Meta-analysis, this study evaluated the current effect sizes of exercise interventions for prenatal depression and refined the best intervention protocols to provide an evidence-based basis for better exercise interventions for clinical caregivers. In terms of effect, exercise can significantly improve antenatal depression. Important factors affecting the impact of exercise intervention for antenatal depression include the type of exercise, frequency, single exercise duration, period, and exercise format in the exercise program. Regarding the best exercise program for exercise intervention for antenatal depression, Yoga and the combination of aerobic exercise were more prominent, and Yoga had the best improvement effect. The use of group exercise 3–5 times a week for 30–60 min for 6–10 weeks is more likely to achieve the desired intervention effect of improving antenatal depression.

### Recommendations

Based on scientific assessment of the physical condition and characteristics of patients with antenatal depression, clinical workers should provide targeted guidance to patients to develop personalized exercise prescriptions and precise interventions and strengthen the multidisciplinary collaboration between theory and practice in this field. Clinical caregivers and health managers should consider various factors such as type, intensity, and duration of exercise to strengthen the safety monitoring of the exercise intervention process to ensure the safety and effectiveness of exercise.

## Data Availability

The data and materials are available from the corresponding author under reasonable request.

## Abbreviations

CNKI: Chinese National Knowledge Infrastructure
MD: mean difference
CI: confidence interval
SD: standard deviation
CES-D: Center for Epidemiologic Studies Depression Scale
ANS: Autonomic Nervous System
VMPFC: ventral medial prefrontal cortex
DA: dopamine
GABA: gamma-aminobutyric acid
